# Phenotypic and genotypic characterization of listeria monocytogenes strains isolated from meat industries

**DOI:** 10.3389/fmicb.2026.1922185

**Published:** 2026-10-08

**Authors:** Despina Vougiouklaki, Markos Dalezios, Konstantina Kyriakou, Marios Papaparaskevas, Marina Siouti, Simen Akkermans, Jan F. M. Impe, Efstathia Tsakali, Dimitra Houhoula

**Affiliations:** 1 Department of Food Science and Technology, Faculty of Food Sciences, University of West Attica, Athens, Greece; 2 Department of Chemical Engineering, BioTeC + Chemical and Biochemical Process Technology and Control, KU Leuven, Gent, Belgium

**Keywords:** antimicrobial susceptibility, whole-genome sequencing, multilocus sequence typing, resistance determinants, plasmids, environmental persistence, food safety

## Abstract

*Listeria monocytogenes* is a major foodborne pathogen of concern for the food industry due to its ability to persist in processing environments and contaminate ready-to-eat foods. This study aimed to characterize phenotypically and genotypically *L. monocytogenes* isolates recovered from meat-processing environments in Greece, with emphasis on antimicrobial susceptibility, resistance determinants, plasmid content, and sequence types. Eighteen environmental isolates were analyzed using broth microdilution MIC testing and Oxford Nanopore whole-genome sequencing followed by *in silico* screening for antimicrobial resistance genes and plasmids. All isolates were susceptible to ampicillin, erythromycin, and trimethoprim–sulfamethoxazole according to EUCAST criteria. For agents categorized by EUCAST as having insufficient evidence (“IE”) for clinical categorization in *Listeria* (moxifloxacin and linezolid), MICs were reported descriptively. Elevated MICs were observed for clindamycin (≥ 8 mg/L) in three isolates and for tetracycline (≥ 16 mg/L) in two isolates, while all isolates showed high fosfomycin MICs (≥ 128 mg/L) and intermediate fusidic acid MICs (4–8 mg/L). Genomic screening identified two acquired resistance genes, *vga (G)* and *tet (M)*, and detected plasmids in a subset of isolates, including plasmid types previously associated with stress tolerance and persistence in food environments. MLST revealed four sequence types (ST87, ST155, ST6, and ST504), including lineages linked to food-processing persistence and clinically relevant clonal complexes. Overall, the findings indicate limited clinically relevant resistance among the examined isolates, while highlighting the contribution of mobile genetic elements and diverse sequence types to environmental fitness and potential public-health risk in meat-processing settings.

## 1 Introduction

In the European Union, human listeriosis is reported less frequently than many other foodborne infections; however, it is associated with high hospitalization and mortality rates, particularly among vulnerable population groups (ECDC, 2022). Consequently, *Listeria monocytogenes* remains a major concern for the food industry. The pathogen is widely distributed in food-production environments and can contaminate animal-derived products, including poultry, beef, and dairy products (Ramaswamy et al., 2007; Orsi and Wiedmann, 2016; Ferreira et al., 2014). *Listeria monocytogenes* is frequently recovered from both food-contact and non-food-contact sites in slaughterhouses and meat-processing facilities, including drains, floors, walls, processing equipment, and other difficult-to-clean niches (Sala et al., 2016). These sites may act as environmental reservoirs and contribute to repeated contamination of raw materials, processing lines, and finished products (Sala et al., 2016; Ferreira et al., 2014). The ability of certain strains to adhere to abiotic surfaces and tolerate refrigeration, nutrient limitation, and sanitation-related stresses may facilitate their long-term survival in food-processing environments (Ferreira et al., 2014; Fagerlund et al., 2022b). Therefore, environmental monitoring of both food-contact and non-food-contact surfaces is important for the early detection and control of persistent contamination.

*L. monocytogenes* can grow under a broad range of environmental conditions, including temperatures from −0.4°C to 45°C, pH values from 4.4 to 9.4, salinity up to 10% NaCl, and water activity as low as 0.90. These physiological characteristics enable survival during refrigeration, salting, and mild acidification and increase the risk of contamination of ready-to-eat foods and food-processing environments (Chan and Wiedmann, 2008; Orsi and Wiedmann, 2016). In addition, its capacity to form biofilms on food-contact surfaces enhances environmental persistence and resistance to sanitation procedures (Finn et al., 2023).

Despite its relatively low incidence in the European Union, listeriosis is associated with severe clinical outcomes, particularly among immunocompromised individuals, pregnant women, neonates, and older adults (ECDC, 2022). This disease burden necessitates continuous surveillance and effective control measures throughout the food-production chain.

Published phenotypic and genomic information on *Listeria monocytogenes* from Greek food-processing environments remains limited, particularly for the meat-processing sector. Previous studies conducted in Greece have mainly examined isolates from other food matrices, including seafood and seafood-market environments, and have reported variable antimicrobial-resistance profiles (Peratikos et al., 2024). In contrast, European surveillance studies have demonstrated that persistent *L. monocytogenes* populations in food-production facilities may carry specific stress-survival determinants, mobile genetic elements, and sequence types that facilitate long-term establishment (Fagerlund et al., 2022b; Unrath et al., 2021).

Antimicrobial resistance in *Listeria* has increasingly been reported worldwide in both clinical and food-associated isolates, although marked geographic variability exists, reflecting differences in antimicrobial use, environmental conditions, and processing practices (Capita and Alonso-Calleja, 2013; Panera-Martínez et al., 2022; Cufaoglu et al., 2021). Resistance development in *Listeria* is mainly associated with acquisition of mobile genetic elements such as plasmids, transposons, and integrative conjugative elements carrying resistance determinants and efflux pumps. Horizontal gene transfer between *Enterococcus, Staphylococcus, Streptococcus*, and *Listeria* species has been documented, facilitating dissemination of resistance genes in food-associated microbial communities (Urban-Chmiel et al., 2022). These elements may also be organized within integrons that enable capture, rearrangement, and expression of resistance genes from diverse sources (Hasan and Al-Harmoosh, 2020).

The present study aimed to characterize phenotypically and genotypically *L. monocytogenes* isolates recovered from meat-processing environments in Greece. Specifically, the study evaluated antimicrobial susceptibility profiles, acquired resistance genes, plasmid content, and sequence-type distribution using MIC testing, whole-genome sequencing, and multilocus sequence typing.

## 2 Materials and methods

### 2.1 Bacterial isolates and sampling

*Listeria* spp. isolates included in this study were retrieved from the culture collection of the Laboratory of Food and Beverage Quality Control and Hygiene, Agricultural University of Athens. Isolates originated from two meat-processing plants (Industry A and Industry B) and were collected during 2024–2025.

More than 100 environmental samples were obtained from multiple sites within the two facilities, including food-contact and non-food-contact surfaces (e.g., processing equipment, walls, floors, and drains). Eighteen *Listeria monocytogenes* isolates were selected for further analysis. Isolates were preserved in tryptone soy broth (TSB) supplemented with 20% (v/v) glycerol and stored at −20°C until use.

### 2.2 Phenotypic characterization

Isolates were recovered from frozen stocks by sub culturing on Agar Listeria according to Ottaviani and Agosti (ALOA) plates (Bioprepare, Keratea, Greece). ALOA is a selective and differential medium for isolation and presumptive identification of *Listeria* spp., enabling differentiation of *L. monocytogenes* based on phospholipase C activity (Jantzen et al., 2006). Plates were incubated at 37 ± 1°C for 24 h. Colonies with typical blue-green coloration and an opaque halo were selected for further testing.

Species identification was confirmed by MALDI-TOF MS using the Vitek MS Prime system (bioMérieux, Marcy l'Etoile, France), according to the manufacturer's instructions. *Escherichia coli* ATCC 8,739 was used as the quality-control strain for MALDI-TOF procedures.

### 2.3 Antibiotic susceptibility testing

Minimum inhibitory concentrations (MICs) were determined using in-house prepared 96-well broth microdilution plates. The following antimicrobials were tested: ampicillin, gentamicin, ciprofloxacin, moxifloxacin, erythromycin, clindamycin, linezolid, vancomycin, teicoplanin, tetracycline, tigecycline, fosfomycin, fusidic acid, rifampicin, and co-trimoxazole (trimethoprim–sulfamethoxazole).

Microdilution plates were prepared and tested according to EUCAST recommendations using Mueller–Hinton Fastidious Broth (Liofilchem, Italy). MIC determination was performed by visual inspection after incubation and was defined as the lowest antimicrobial concentration showing no visible bacterial growth. All isolates were tested in duplicate.

MIC interpretation followed EUCAST clinical breakpoints and guidance for *Listeria monocytogenes* (EUCAST v16.0; accessed from the EUCAST breakpoint tables). For agents categorized by EUCAST as “IE” (insufficient evidence), MICs were reported descriptively without classification as susceptible or resistant. *Streptococcus pneumoniae* ATCC 49,619 was used for quality control.

### 2.4 Genotypic characterization of listeria monocytogenes isolates

#### 2.4.1 Serogrouping of isolates

The isolates were previously identified and classified as *Listeria monocytogenes* or non-identified *Listeria* spp. by the Laboratory of Food and Beverage Quality Control and Hygiene, Agricultural University of Athens. Serogrouping of the 18 confirmed *L. monocytogenes* isolates was performed according to the method described by Lachtara et al. (2021). The primer sequences used in the PCR were specific for L. monocytogenes serogroup determination: prs and lmo0737 for IIa; prs and ORF2819 for IIb; prs, lmo1118 and lmo0737 for IIc; and prs, ORF2110 and ORF2819 for IVb (Doumith et al., 2004).

#### 2.4.2 DNA extraction and quantification

Actively growing cells were obtained by subculturing isolates on ALOA plates under aseptic conditions. Genomic DNA was extracted using the QIAamp^®^ Fast DNA Stool Mini Kit (QIAGEN), following the manufacturer's instructions with minor modifications to optimize bacterial lysis. DNA concentration and quality were assessed fluorometrically using the Quantus™ Fluorometer (Promega) and QuantiFluor^®^ ONE dsDNA dye. DNA extracts with concentrations >15 ng/μL were stored at −20°C until sequencing.

#### 2.4.3 Whole-genome sequencing and genomic analysis

Whole-genome sequencing was performed using the Ligation Sequencing g DNA—Native Barcoding Kit 96 V14 (SQK-NBD114.96; Oxford Nanopore Technologies, Oxford, UK), according to the manufacturer's protocol. Sequencing runs were performed on new MinION flow cells (R10.4.1) using a MinION Mk1C device with MinKNOW software (v21.11.7). Basecalling was conducted using Guppy (v5.1.13) in high-accuracy mode, generating FASTQ files.

High-quality reads were uploaded to the cloud-based BUGSEQ platform for automated analysis, including taxonomic assignment at species/strain level and detection of antibiotic resistance genes (ARGs), plasmids, virulence-associated genes, and mutations linked to antimicrobial resistance. Outputs were subsequently curated and summarized using standard bioinformatics and statistical tools.

## 3 Results

### 3.1 Characterization of isolates and sampling sites

From more than 100 environmental samples collected from meat and meat-product processing facilities, 18 isolates were selected and previously confirmed as *Listeria monocytogenes*. These isolates were subsequently assigned to their corresponding serogroups. Serogroup distribution profiles are presented in Table 1.

**Table 1:** Final serogroups profiles (*N* = 18).

Isolate	Serogroup	*N*	%
LM1	1/2b	5	27.8
LM2	1/2a	5	27.8
LM3	1/2a	5	27.8
LM4	1/2a	5	27.8
LM5	1/2c	5	27.8
LM6	1/2b	5	27.8
LM7	4b	3	16.7
LM8	1/2b	5	27.8
LM9	4b	3	16.7
LM10	1/2a	5	27.8
LM11	1/2c	5	27.8
LM12	1/2c	5	27.8
LM13	1/2c	5	27.8
LM14	4b	3	16.7
LM15	1/2c	5	27.8
LM16	1/2b	5	27.8
LM17	1/2b	5	27.8
LM18	1/2a	5	27.8

All isolates originated from food-contact and non-food-contact surfaces within meat-processing environments. Sampling sites included the meat scraping shelf (5.6%), transport carts (16.7%), wall skirting surfaces (5.6%), Teflon-coated meat-cutting surfaces (5.6%), drains (38.9%), tumbling barrel surfaces (27.8%), and meat-cutting benches (5.6%).

Serogroups 1/2a, 1/2c, and 1/2b were equally represented (n = 5; 27.8% each), whereas serogroup 4b was less frequently detected (*n* = 3; 16.7%).

### 3.2 Antibiotic susceptibility testing

MIC values for the 18 *L. monocytogenes* isolates are presented in Table 2. All isolates were susceptible to ampicillin, erythromycin, and co-trimoxazole according to EUCAST criteria. For moxifloxacin and linezolid, EUCAST currently classifies evidence as insufficient (“IE”) for clinical categorization in *Listeria*; therefore, MICs were reported descriptively. In general, moxifloxacin MICs were low (0.5–1 mg/L), whereas linezolid MICs clustered at 2–4 mg/L.

**Table 2:** MIC results of the 18 isolates.

No	Serogroup	Amp^*^	Mfx^*^	Lzd^*^	Ery^*^	Sxt^*^	Gen	Cip	Cli	Va	Tec	Tet	Tgc	Fos	Fa	Ra
LM1	1/2b	≤ 1	1	4	≤ 0.25	≤ 0.06	≤ 0.5	1	2	≤ 0.5	≤ 0.5	≤ 1	≤ 0.12	≥128	4	≤ 0.5
LM2	1/2a	≤ 1	1	4	≤ 0.25	≤ 0.06	≤ 0.5	1	2	≤ 0.5	≤ 0.5	≤ 1	≤ 0.12	≥128	4	≤ 0.5
LM3	1/2a	≤ 1	1	4	≤ 0.25	≤ 0.06	≤ 0.5	1	2	≤ 0.5	≤ 0.5	≤ 1	≤ 0.12	≥128	4	≤ 0.5
LM4	1/2a	≤ 1	1	4	≤ 0.25	≤ 0.06	≤ 0.5	1	≤ 0.25	≤ 0.5	≤ 0.5	≤ 1	≤ 0.12	≥128	8	≤ 0.5
LM5	1/2c	≤ 1	1	4	≤ 0.25	≤ 0.06	≤ 0.5	1	≥8	≤ 0.5	≤ 0.5	≥16	≤ 0.12	≥128	8	≤ 0.5
LM6	1/2b	≤ 1	0.5	4	≤ 0.25	≤ 0.06	≤ 0.5	≤ 0.5	2	≤ 0.5	≤ 0.5	≤ 1	≤ 0.12	≥128	4	≤ 0.5
LM7	4b	≤ 1	1	2	≤ 0.25	≤ 0.06	≤ 0.5	1	≤ 0.25	≤ 0.5	≤ 0.5	≤ 1	≤ 0.12	≥128	8	≤ 0.5
LM8	1/2b	≤ 1	1	4	≤ 0.25	≤ 0.06	≤ 0.5	1	≤ 0.25	≤ 0.5	≤ 0.5	≤ 1	≤ 0.12	≥128	4	≤ 0.5
LM9	4b	≤ 1	1	2	≤ 0.25	≤ 0.06	≤ 0.5	1	2	≤ 0.5	≤ 0.5	≤ 1	≤ 0.12	≥128	8	≤ 0.5
LM10	1/2a	≤ 1	1	4	≤ 0.25	≤ 0.06	≤ 0.5	1	≥8	≤ 0.5	≤ 0.5	≤ 1	≤ 0.12	≥128	4	≤ 0.5
LM11	1/2c	≤ 1	1	4	≤ 0.25	≤ 0.06	≤ 0.5	1	≤ 0.25	≤ 0.5	≤ 0.5	≤ 1	≤ 0.12	≥128	8	≤ 0.5
LM12	1/2c	≤ 1	1	4	≤ 0.25	≤ 0.06	≤ 0.5	1	≥8	≤ 0.5	≤ 0.5	≤ 1	≤ 0.12	≥128	8	≤ 0.5
LM13	1/2c	≤ 1	1	4	≤ 0.25	≤ 0.06	≤ 0.5	1	≤ 0.25	≤ 0.5	≤ 0.5	≥16	≤ 0.12	≥128	4	≤ 0.5
LM14	4b	≤ 1	1	4	≤ 0.25	≤ 0.06	≤ 0.5	1	2	≤ 0.5	≤ 0.5	≤ 1	≤ 0.12	≥128	4	≤ 0.5
LM15	1/2c	≤ 1	1	4	≤ 0.25	≤ 0.06	≤ 0.5	1	2	≤ 0.5	≤ 0.5	≤ 1	≤ 0.12	≥128	8	≤ 0.5
LM16	1/2b	≤ 1	1	4	≤ 0.25	≤ 0.06	≤ 0.5	1	1	≤ 0.5	≤ 0.5	≤ 1	≤ 0.12	≥128	8	≤ 0.5
LM17	1/2b	≤ 1	1	4	≤ 0.25	≤ 0.06	≤ 0.5	1	2	≤ 0.5	≤ 0.5	≤ 1	≤ 0.12	≥128	8	≤ 0.5
LM18	1/2a	≤ 1	0.5	4	≤ 0.25	≤ 0.06	≤ 0.5	≤ 0.5	≤ 0.25	≤ 0.5	≤ 0.5	≤ 1	≤ 0.12	≥128	4	≤ 0.5

Amp, ampicillin; Mfx, moxifloxacin; Lzd, linezolid; Ery, erythromycin; Sxt, co-trimoxazole; Gen, gentamicin; Cip, ciprofloxacin; Cli, clindamycin; Va, vancomycin; Tec, teicoplanin; Tet, tetracycline; Tgc, tigecycline; Fos, fosfomycin; Fa, fucidic acid; Ra, rifampicin. ^*^EUCAST included agents. EUCAST V16.0 (2026) guidelines and susceptibility criteria: Amp S ≤ 1 R >1, Mfx IE, Lzd IE, Ery S ≤ 1 R >1 and Sxt S ≤ 0.06 R >0.06 (IE: insufficient evidence that the antibiotic can be used for Listeria infections). For the rest of the antibiotics there are no guidelines and susceptibility criteria.

For antimicrobials without EUCAST breakpoints for *Listeria*, most isolates exhibited low MICs for gentamicin, ciprofloxacin, vancomycin, teicoplanin, tigecycline, and rifampicin. Elevated clindamycin MICs (≥8 mg/L) were observed in isolates LM5 (serogroup 1/2c), LM10 (1/2a), and LM12 (1/2c). Elevated tetracycline MICs (≥16 mg/L) were observed in isolates LM5 (1/2c) and LM13 (1/2c). All isolates exhibited high fosfomycin MICs (≥128 mg/L) and intermediate fusidic acid MICs (4–8 mg/L).

Regarding other guidelines, CLSI provides Listeria breakpoints in the M45 document, where, regarding the antibiotics tested here, CLSI uses the Ampicillin 2 mg/L breakpoint and the Co-trimoxazole 0.5 mg/L breakpoint, and in that respect all our isolates were also classified as susceptible.

### 3.3 Antimicrobial resistance genes and genotypic prediction of antimicrobial resistance

Genomic screening using BUGSEQ did not detect resistance predictors for ampicillin, penicillin, meropenem, or trimethoprim–sulfamethoxazole in any of the 18 *L. monocytogenes* isolates. Two acquired antimicrobial resistance genes were detected across the dataset: *vga(G)* and *tet(M)*.

### 3.4 Multilocus sequence typing (MLST)

Isolates classified as *Listeria monocytogenes* in the Top Taxa analysis and exhibiting >95% relative abundance were subjected to multilocus sequence typing (MLST) to determine their sequence types (STs). In heterogeneous samples where the full set of housekeeping loci could not be recovered, ST assignment was not performed. For such cases, the BIGSdb-Pasteur database was used to identify the closest matching sequence types based on the available allelic profiles. The detected sequence types (STs) included ST155, ST504, ST87, and ST6. Overall, among the typed isolates (n = 9), ST87 and ST155 were the most prevalent (each 33.3%), followed by ST6 (22.2%) and ST504 (11.1%) (Table 3).

**Table 3:** Distribution of isolates across the identified sequence types (STs) based on MLST analysis.

Sequence type (ST)	Isolates (IDs)	*n*	% (of typed isolates, *n* = 9)
ST87	LM8, LM14, LM18	3	33.3
ST155	LM2, LM3, LM10	3	33.3
ST6	LM7, LM9	2	22.2
ST504	LM4	1	11.1
Total		9	100.0

### 3.5 Identification and characterization of plasmids

Genomic analysis identified plasmids in four isolates (LM10, LM12, LM14, and LM16). The LM10 isolate contained one plasmid assigned to the AG939 cluster. The LM12 isolate harbored two plasmids, assigned to the AC309 and AG939 clusters. The LM14 isolate harbored two plasmid subtypes: AG939-subAQ995 and AC310-subAL449. The LM16 isolate carried plasmid subtype AL445, belonging to the AC309 cluster. No antimicrobial resistance markers were detected.

## 4 Discussion

The present study investigated the phenotypic and genotypic characteristics of *Listeria monocytogenes* isolates originating from meat-processing environments in Greece, focusing on antimicrobial susceptibility, resistance determinants, plasmid content, and sequence types. Overall, the isolates exhibited low levels of clinically relevant antimicrobial resistance, consistent with previous reports indicating that food-associated *L. monocytogenes* generally remains susceptible to first-line anti-listerial agents (Wagner et al., 2020; Sousa et al., 2024).

All isolates were susceptible to ampicillin, erythromycin, and trimethoprim–sulfamethoxazole according to EUCAST criteria, confirming the continued effectiveness of recommended therapeutic options for listeriosis. No multidrug-resistant (MDR) phenotypes were detected. Similar susceptibility patterns have been reported in European food and environmental isolates, in contrast to studies from other regions where increased resistance and MDR have been described (Peratikos et al., 2024; Silva et al., 2024; Sołtysiuk et al., 2025). The comparatively low resistance observed here suggests limited antimicrobial selective pressure in the examined meat-processing environments or effective sanitation practices restricting dissemination of resistant clones.

Elevated MIC values for clindamycin and tetracycline were observed in four isolates (LM5, LM10, LM12, and LM13). Acquired resistance genes *vga (G)* and *tet (M)* were also detected in the dataset; however, direct isolate-level association between phenotypic resistance and specific resistance determinants could not be conclusively established. The *vga (G)* determinant encodes an ABC-F ribosomal protection protein mediating reduced susceptibility to lincosamides, streptogramin A, and pleuromutilins (Bechtel and Gibbons, 2021; Gana et al., 2024), whereas *tet (M)* is one of the most widely distributed tetracycline resistance genes associated with mobile genetic elements and horizontal transfer in food-associated bacteria (Haubert et al., 2016; Ji et al., 2023; Anupama et al., 2025). Their occurrence in a subset of isolates supports sporadic acquisition events within processing environments. Nevertheless, the limited distribution of these genes and absence of MDR profiles indicate that acquired resistance remains uncommon in the studied facilities.

All isolates displayed high MICs to fosfomycin and intermediate MICs to fusidic acid and linezolid. Interpretation of these findings should be cautious, as EUCAST breakpoints for *Listeria* are not established for these agents. High fosfomycin MICs are frequently reported in *L. monocytogenes* and are considered intrinsic or of limited clinical relevance, reflecting permeability and metabolic characteristics rather than acquired resistance (Rodríguez-Melcón et al., 2021). Similarly, intermediate linezolid and fusidic-acid MICs in the absence of known resistance determinants likely represent natural variability rather than clinically meaningful resistance. This highlights the importance of integrating phenotypic and genomic data and avoiding overinterpretation of MIC values for antibiotic–species combinations lacking validated breakpoints.

Genomic analysis identified plasmids belonging to the AC309, AC310, and AG939 clusters in four isolates. Related plasmids have previously been described in food-associated *Listeria monocytogenes* and have been linked mainly to stress adaptation and environmental persistence. AC309-related plasmids have been reported to carry genes associated with tolerance to disinfectants and heavy metals (Voronina et al., 2023), while pLM58-related plasmids carrying the heat-shock gene *clpL* have been shown to enhance survival under heat stress (Pöntinen et al., 2017). AC310-subAL449-like plasmids have also been associated with improved survival under high-salinity and acidic conditions (Hingston et al., 2019; Parra-Flores et al., 2022). In addition, AG939-related plasmids have been linked in previous studies to persistence-associated traits and have been reported to carry resistance determinants such as *tet (M)* and *vga (G)* (Hingston et al., 2019). However, these functional associations were not experimentally evaluated in the present study, and the detection of related plasmid clusters should not be interpreted as direct evidence of enhanced persistence or antimicrobial resistance in the examined isolates. MLST analysis identified ST87, ST155, ST6, and ST504, sequence types that have previously been reported in food-processing environments, food products, and clinical cases. ST155 has frequently been detected among food-associated and processing-environment isolates and has been linked in previous studies to prolonged environmental circulation, although strains within this sequence type may show heterogeneous virulence characteristics (Wagner et al., 2020). ST504 has been reported less frequently but has been recovered from meat-associated environments and invasive human infections, suggesting that its occurrence is not restricted to a single ecological niche (Sousa et al., 2024). ST6 belongs to a clinically important lineage repeatedly implicated in severe listeriosis cases and foodborne outbreaks, including outbreaks associated with ready-to-eat products (Slavinska et al., 2025). ST87 has also been reported in food and clinical settings and has been associated with serious human infections in previous epidemiological investigations (Cabal et al., 2019). However, MLST identifies broad genetic lineages and does not by itself establish persistence, virulence potential, or epidemiological relatedness at the isolate level (Cooper and Feil, 2004; Cortes et al., 2020). Therefore, cgMLST or SNP-based analysis, together with isolate-specific characterization of virulence and stress-survival determinants, would be required to define the precise public-health significance of the isolates examined here.

Compared with previous international studies reporting high resistance rates and MDR among food and environmental *Listeria*, the present isolates exhibited markedly lower resistance frequencies. For example, high clindamycin resistance has been reported in Greek seafood isolates (Peratikos et al., 2024), whereas MDR rates exceeding 80% have been described in other regions (Silva et al., 2024; Sołtysiuk et al., 2025). Differences in antimicrobial usage, sanitation practices, and methodological approaches—particularly non-standardized disk diffusion criteria—likely contribute to this variability. The use of EUCAST MIC-based interpretation in the present study provides more conservative and clinically relevant resistance assessment.

Importantly, the absence of genotypic predictors for clinically relevant antibiotics despite occasional elevated MICs indicates that phenotypic variation may arise from mechanisms not captured by current databases, such as regulatory changes or point mutations. This emphasizes the need for combined phenotypic and genomic surveillance to accurately evaluate antimicrobial resistance in *Listeria* populations.

A major limitation of the present study is the relatively small number of *Listeria monocytogenes* isolates analyzed and their origin from only two meat-processing facilities. Therefore, the observed antimicrobial susceptibility profiles, resistance determinants, plasmid distribution, and sequence types should not be considered representative of the broader epidemiology of *L. monocytogenes* in Greek meat-processing environments. Rather, the findings should be interpreted as preliminary, facility-specific surveillance data. Future studies should include larger isolate collections from multiple facilities and geographical regions and should incorporate longitudinal sampling to better assess the distribution, persistence, and epidemiological significance of specific clones.

The added value of the present study lies in the combined phenotypic and genomic characterization of *Listeria monocytogenes* isolates from an underrepresented food-production sector in Greece. Epidemiological data regarding *Listeria monocytogenes* and other Listeria species in fresh-meat processing environments in Greece are currently unavailable but unlike previous Greek reports focusing mainly on other food matrices, this dataset integrates MIC testing with resistance-gene screening, plasmid characterization, serogrouping, and MLST. The detection of both persistence-associated and clinically relevant sequence types, together with plasmid clusters linked in previous studies to environmental stress tolerance, provides a preliminary genomic baseline for Greek meat-processing facilities. Future studies should include the characterization of non-monocytogenes Listeria species, as well as the investigation of antimicrobial resistance, virulence, persistence, and biofilm-associated determinants, to obtain a more comprehensive understanding of the epidemiological situation in Greece. However, these observations should be interpreted as facility-specific findings rather than as representative of the national epidemiology of *L. monocytogenes*.

## 5 Conclusion

The 18 *Listeria monocytogenes* isolates recovered from two Greek meat-processing facilities showed limited clinically relevant antimicrobial resistance and remained susceptible to ampicillin, erythromycin, and trimethoprim–sulfamethoxazole according to EUCAST criteria. Elevated clindamycin and tetracycline MICs were detected in a small number of isolates, while the acquired resistance genes *vga (G)* and *tet (M)* were identified in the dataset. Plasmids associated mainly with stress tolerance and environmental persistence were detected in a subset of isolates. MLST identified ST87, ST155, ST6, and ST504, demonstrating the presence of both persistence-associated and clinically relevant lineages. These findings provide preliminary phenotypic and genomic data on *L. monocytogenes* from Greek meat-processing facilities and support the use of combined antimicrobial susceptibility testing and genomic surveillance for monitoring contamination and persistent strains. However, due to the limited number of isolates and facilities examined, the findings should be interpreted as facility-specific and not as representative of the national epidemiology of *L. monocytogenes*.

## Data Availability

The original contributions presented in the study are available: https://doi.org/10.5281/zenodo.23035497.
